# Business Process Compliance Using Reference Models of Law

**DOI:** 10.1007/978-3-030-45234-6_19

**Published:** 2020-03-13

**Authors:** Hugo A. López, Søren Debois, Tijs Slaats, Thomas T. Hildebrandt

**Affiliations:** 8 University of Paderborn, Paderborn, Germany; 9 ICREA, Open University of Catalonia, Barcelona, Spain; 10 Software, Data, People & Society Section, Department of Computer Science, Copenhagen University, Copenhagen, Denmark; 11 Computer Science Department, IT University of Copenhagen, Copenhagen, Denmark; 12 DCR Solutions A/S, Copenhagen, Denmark

**Keywords:** Formal Models of Law, Dynamic Condition Response (DCR) graphs, Compliance Checking, Process Calculi, Refinement

## Abstract

Legal compliance is an important part of certifying the correct behaviour of a business process. To be compliant, organizations might hard-wire regulations into processes, limiting the discretion that workers have when choosing what activities should be executed in a case. Worse, hard-wired compliant processes are difficult to change when laws change, and this occurs very often. This paper proposes a model-driven approach to process compliance and combines a) reference models from laws, and b) business process models. Both reference and process models are expressed in a declarative process language, The Dynamic Condition Response (DCR) graphs. They are subject to testing and verification, allowing law practitioners to check consistency against the intent of the law. Compliance checking is a combination of alignments between events in laws and events in a process model. In this way, a reference model can be used to check different process variants. Moreover, changes in the reference model due to law changes do not necessarily invalidate existing processes, allowing their reuse and adaptation. We exemplify the framework via the alignment of laws and business rules and a real contract change management process, Finally, we show how compliance checking for declarative processes is decidable, and provide a polynomial time approximation that contrasts NP complexity algorithms used in compliance checking for imperative business processes. All-together, this paper presents technical and methodological steps that are being used by legal practitioners in municipal governments in their efforts towards digitalization of work practices in the public sector.

## 1 Introduction

Ensuring that business processes comply with applicable laws and regulations has been a central concern with the arrival of regulatory technologies (RegTech), and bring together different disciplines ranging from legal theory to computer science. We understand compliance as the *"act/process to ensure that business operations, processes, and practices are in accordance with prescriptive (often legal) documents"* [15]. Checking compliance requires ways to compare artefacts coming from very different domains: the legal domain and the process domain. On the one hand, business processes have as a main criteria the fulfilment of a business goal. On the other hand, processes operate within a regulated context, that sets certain limitations on how to achieve the goals, and defines responsibilities for actors involved. In the public sector, being non-compliant is not an option, as regulations determine the rights and obligations of their citizens. In the private sector, the risk of being non-compliant equates to possible hefty fines for the organization[4].

Linking laws and processes have several challenges: First, how can we formally interpret ambiguous regulations written in natural language? Second, how to pair that formal interpretation of the law against a business process? Third, how to reuse legal specifications in different process domains?, and fourth, what will happen with compliance when the laws change? *Compliance checking* refers to the verification procedure that compares regulations and processes: In its most simple form, compliance checking can be expressed as the following problem: given a formal specification of a law $L$ and a business process $P$, we say that the process is compliant if 1. Every action that $P$ does is in accordance to the permissions allowed by $L$, and 2. Every execution of $P$ meets the set of obligations established by $L$, and 3. Executions of $P$ don't do anything prohibited by $L$. In any other case we will say that the process is not compliant.

In this paper we focus on the compliance checking problem from a modelling/programming language perspective. First, we explore how declarative process languages can describe the set of requirements expressed in legal documents. The challenge is both at the level of language expressiveness (can the language express the intended semantics of a legal text?), as well as understandability (can a non-expert understand the specification?). Second, we look at the process dimension: can we have a general framework that considers different process artefacts? Third, we look at the alignment between the legal and the process dimension: Can we provide an efficient algorithm to compute whether a process is compliant with the legislation?

In [20], a taxonomy of the requirements needed to formally express laws was presented. Overall, a formal language that expresses legal requirements should be able to describe what can be done (*permissions*), what must be done (*obligations*), and what should not happen (*violations*). Moreover, these so-called deontic constraints are *effectful* (e.g.: an obligation might grant certain permissions, e.g. "you must pay for delivery, but when you do so, you may decide whether to pay now or upon delivery" and vice-versa, a permission may impose certain obligations, e.g. "you may park here if you pay later"). The content of the laws might also influence the choice of the language. Laws might describe

[4] https://www.theverge.com/2019/1/21/18191591/google-gdpr-fine-50-million-euros-data-consent-cnil constraints related to the control flow, temporal information, data, or resource constraints [39]. Finally, the language of choice should be able to describe *defeasible conditions* [18], that is, when parts of the law become irrelevant, and are superseded by other parts.

Compliance checking requires a formal representation of business goals and processes. Such a representation traditionally takes the shape of traces (c.f.: event-logs) at run-time, and of *imperative* process models at design-time. In the imperative paradigm, languages such as BPMN [35] and UML Activity Diagrams [34] describe processes as activities and composition operators that prescribe *how* the flow in the activities executed in the process. Rules and laws are not first-class citizens in imperative models, and they need to be encoded as annotations in the process language [13], or paired with additional languages, such as BPMN-Q [4]. In contrast, declarative process models focus in the description of circumstantial information of processes (e.g.: the *why* of the process). Languages such as Declare [37] and Dynamic Condition Response (DCR) Graphs [10, 22] are some exponents of these types of languages. They describe a process as a set of constraints between activities which can be translated to specific business rules or goals. Their semantics is usually characterised by either mapping the declarative model to a flow-based model (e.g. transition systems), or by introducing an operational semantics that reasons over the state of the different constraints and/or activities of the model.

The objective of this paper is two-fold. First, it explores whether existing declarative process languages are expressive enough to formalise regulations; second, it introduces compliance checking via declarative processes. The DCR graphs process notation has been developed for the formalisation and digitalisation of collaborative, adaptive case management processes. The visual notation is both supported by a range of formal techniques, and serves as the formal base for the industrial (www.dcrgraphs.net) modelling and simulation tool. In contrast to Declare, the DCR graphs technology has been succesfully employed in major industrial case management systems, and at the moment it supports 70% of the Danish Central Government institutions[5]. DCR graphs have been extended to include both data [43], time [5, 24], sub-processes [10], and choreographies [25]. In the present paper we consider the core notation with time, which is expressive enough to represent both regular and omega-regular languages [10] as well as so-called true concurrency [9]. In this work we only focus on laws describing control-flow and temporal constraints, leaving data, resource constraints or inter-law dependencies for future work.

Our approach for process compliance can be summarised as follows: both the legal domain and the business/organisational domain are defined as independent DCR graphs, and compliance checking is reduced to process refinement. These two independent models allow for a separation of concerns on what is legal and what is business/organisational requirements and goals, and it eases compliance checking when either laws or organisational processes change. It is worth to point out that at its core, the choice of a process language can be replaced to any

[5] https://www.kmd.dk/indsigter/fleksibilitet-og-dynamisk-sagsbehandling-i-staten existing process language (including imperative ones), as compliance checking is mainly defined over traces. Changes in regulations might affect existing running processes: the typical example is governmental case work, where processes need to be revised every time a new regulation is signed. In addition, organisational changes or process optimisation efforts might modify a business process in a way that stops being compliant with existing laws. Finally, the separation of the legal and business domains supports different stages of the compliance life cycle: designing new processes that are compliant with the laws (e.g.: *Compliance-by-Design* (CbD) [14]), as well as the verification of existing or mined process models [33] becomes possible.

**Contributions** This paper presents the first compliance framework for declarative process models that 1) can represent safety and omega-regular liveness properties, 2) is supported by industrial design and simulation tools, and 3) is currently in use in the digitalization strategies of municipal governments, and 4) allows for a separation of concerns between what is legal and what is process-specific. Thanks to having the *same formal language* for laws and business processes, we can use efficient verification techniques based on process refinement, This comes in contrast to approaches based in annotated imperative business processes, where the complexity of compliance checking belongs to the non-polynomial complexity class [45].

**Document Structure** Section 2 introduces the compliance framework. Section 3 presents DCR graphs, and illustrates its use on a case study. Section 4 explains the construction of reference models. Section 5 describes our compliance checking technique. Results from validation with organizations are documented in Section 6. Related work is compared in 7. We conclude in Section 8.

## 2 Regulatory Compliance Framework

The overall components of our compliance framework are described in Fig. 1. It shows the interactions between two different type of roles: The compliance officer, with a background in law, identifies the applicable regulations, and for each law she generates a reference model. Laws might be abstract, e.g.: *"Any information relating to an identified or identifiable natural person ('data subject')"* (Art. 4 in GDPR [7]). Consequently, the officer might need to combine the law with implementation acts (e.g. the Danish Data Protection Act [8]). In this way, the specification must narrow down ambiguities such as: "What corresponds to any information"?, "in which ways will the process identify a person"? or "who constitutes a natural person"? While the disambiguation process is mostly a manual processes that depends on the expertise of the compliance officer, computer support might provide help in the elicitation phase. Dual-coding tools support lawyers in the generation of formal specifications [29], and NLP techniques can be used to speedup the identification of process-related information [30]. The output will be a collection of reference models, each of them describing a law. Each model describe roles, rights, obligations, and the relations between them.

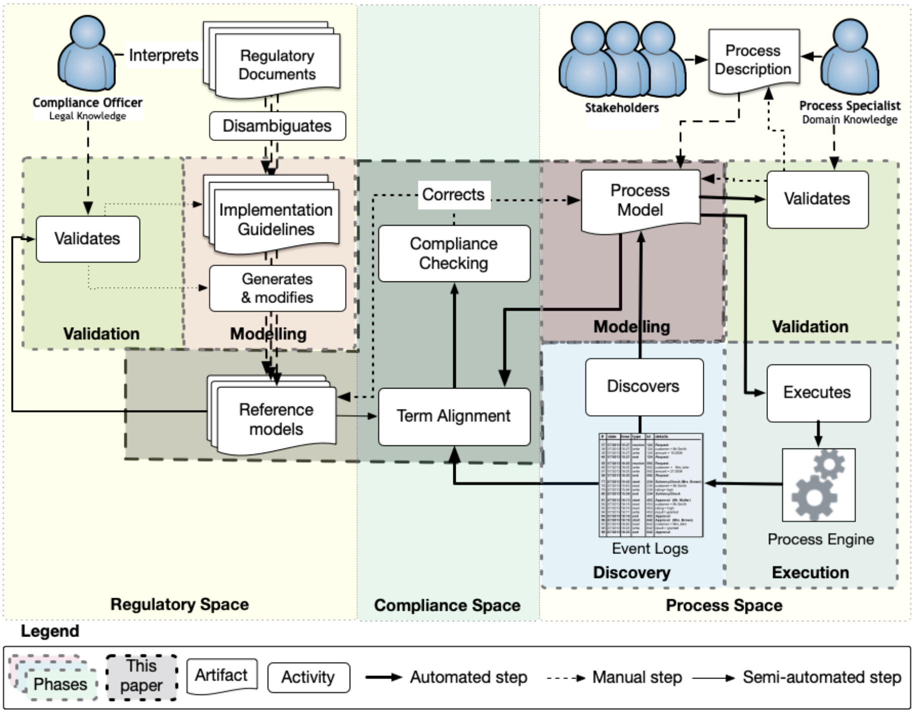

**Fig. 1.** Compliance Framework

Compliance checking assumes the existence of a process. This can be elicited from stakeholders via standard techniques [12] or, if the process already exists, via process mining [33]. Process models contain the activities performed, roles, and resource information (time & data) used. Alternatively, one can consider disregarding process discovery and perform compliance checking directly over event logs, as in classical process conformance approaches [1].

Both models and process models are subject to verification and validation phases. Scenario replays, reachability and deadlock-livelock checkers provide guarantees that both structural properties of the models are preserved.

The last dimension revolves compliance, and it constitutes the core of this paper. Since reference models are specific to a given regulation, they need to be instantiated in terms of the business process. This requires the alignment between events identified in the reference model, and activities in the business process. Compliance checking is then reduced to trace refinement: all traces in the process model are a subset of the traces in the reference model.

The separation between reference and compliance models allows for modular verification. When laws and processes change, their models can be changed separately, only needing to revise the alignment between events and activities.

| | | | |
|---|---|---|---|
| $T, U ::= e \xrightarrow{t_0}\!\bullet f$ | condition | $\mid e \bullet\!\xrightarrow{t_\omega} f$ | response |
| $\mid e \rightarrow\!+ f$ | inclusion | $\mid e \rightarrow\!\% f$ | exclusion |
| $\mid e \rightarrow\!\diamond f$ | milestone | $\mid T \parallel U$ | parallel composition |
| $\mid 0$ | unit | | |
| $M, N ::= M, e : \Phi \mid \epsilon$ | marking | $\Phi ::= (h, i, p)$ | event state |
| $\lambda ::= \lambda, e : l \mid \epsilon$ | labelling | $h ::= \mathsf{f} \mid t_0$ | (h)appened $t_0$ ticks in the past |
| $P, Q ::= [M]\ \lambda T$ | process | $i ::= \mathsf{f} \mid \mathsf{t}$ | (i)ncluded |
| | | $p ::= \mathsf{f} \mid 0 \mid t_\omega$ | (p)ending deadline |
| $t_0 \in \mathbb{N} \cup \{0\}$ | 0-time | $t_\omega \in \mathbb{N} \cup \{\omega\}$ | $\omega$-time |

**Fig. 2.** DCR Processes Syntax.

## 3 DCR Graphs

In this section, we recall the syntax and semantics of Dynamic Condition Response (DCR) processes. We use the core term-based definition with time, without bound events and subprocesses, following the original presentation in [5].

We assume a fixed universe of *events* $\mathcal{E}$ ranged over by $e, f$ with a special symbol $\mathsf{tick} \notin \mathcal{E}$. A DCR process $[M]\ T$ comprises a *marking* $M$, a *term* $T$. Its syntax is given in Figure 2.

A *term* represents a process model consisting of events (which may be activities, tasks, or the identification of the state of affairs) and their relations. In a DCR graph, events are the nodes and relations are the arcs. A *marking* represents the current state of a process by specifying for every event the event state (whether the event previously happened, is currently included, and/or is pending). A *process* is then represented by the process model (a term) and its current state (a marking). Relations can take the following shape:

- Condition $e \xrightarrow{t}\!\bullet f$: It defines a *prohibition*, or a precondition for $f$. Before $f$ can occur, $e$ must have happened at least $t$ time units ago, or $e$ must have been excluded. In the case that $t = 0$, we simply write $e \rightarrow\!\bullet f$.
- Response $e \bullet\!\xrightarrow{t} f$: It defines an *obligation* for $e$. If $e$ has happened, then $f$ must occur within $t$ time units, or be excluded. In the case $t = \omega$, this will be treated as eventually in LTL, that is, not bounded by any time constraint. For such a case we can simply write $e \bullet\!\rightarrow f$.
- Dynamic Inclusion $e \rightarrow\!+ f$: It defines *relevance* of an event. After executing event $e$, event $f$ is included among the possible actions to take. Notice that the inclusion of $f$ does not deem its necessity (captured by a response).
- Dynamic Exclusion $e \rightarrow\!\% f$: It defines *irrelevance* of an event. The result of executing $e$ is that event $f$ becomes excluded. Moreover, all conditions $f \rightarrow\!\bullet g$ and milestones $f \rightarrow\!\diamond g$ are ignored (unless $f$ is included again).
- Milestone $e \rightarrow\!\diamond f$: A reaction chain. Initially $f$ is included among the possible actions, but if $e$ becomes pending, then $f$ cannot occur until $e$ has occurred.

Finally, term 0 denotes the null process. Note that it is possible to specify a relation twice, e.g., $e \rightarrow\% f \parallel e \rightarrow\% f$; this duplication has no additional effect.

All relations refer to a marking $M$, a finite map from events to triples of variables $(h, i, p)$, referred to as the *event state* and indicating whether or not the event previously (h)appened, is currently (i)ncluded, and/or is (p)ending. A pending event represents an unfulfilled obligation, and the values it can take denote whether the event is not pending ($p = \mathsf{f}$), it has a finite deadline ($p \in \mathbb{N} \cup \{0\}$), or it should be eventually executed ($p = \omega$). We write markings as finite lists of pairs of events and event states, e.g. $e_1 : \Phi_1, \ldots, e_k : \Phi_k$ but treat them as maps, writing $\mathsf{dom}(M)$ and $M(e)$, and understand $M, e : \Phi$ to be undefined when $e \in \mathsf{dom}(M)$. The *free events* $\mathsf{fe}(T)$ of a term $T$ is simply the set of events appearing in it.

With respect to the original presentation [5], our syntax extends the process definition with labels. Labelling $\lambda$ defines a total function from events to labels. However, we often omit the labelling function, as it rarely changes, writing $[M]\ T$ instead of $[M]\ \lambda T$ . We assume that event labels are unique, e.g.: if $e, f \in \mathsf{fe}(T)$ then $\lambda(e) \neq \lambda(f)$ or $e = f$, therefore, $\lambda$ has an inverse, which we will denote by $\lambda^{-1}$. A substitution $\sigma = \{e_1, \ldots, e_n / f_1, \ldots, f_n\}$ maps each event $e_i$ and replaces it with $f_i$, being $1 \leq i \leq n$ and $e_i$ pairwise distinct. The application of $\sigma$ to a process term $T$ is denoted by $T\sigma$, and it applies similarly for markings and for processes, being $([M]\ T)\sigma = [M\sigma]\ T\sigma$. We require of a process $P = [M]\ \lambda T$ that $\mathsf{fe}(T) \subseteq \mathsf{dom}(M) = \mathsf{dom}(\lambda)$, and so define $\mathsf{fe}(P) = \mathsf{dom}(M)$. The *alphabet* $\mathsf{alph}(P)$ is the set of labels of its free events.

*Example 3.1.* We use a contract change management process from the construction industry as our running example. The process model in Fig. 3 has been extracted from structured interviews with domain specialists, and then validated in a workshop. We will focus on the most salient aspects of the process, and direct to [2] for the complete specification. The process includes three significant roles: a subcontractor, a project manager and a trade package manager (TPM) –external to the organization–, collaborating via a document management system. The process starts when the subcontractor notices that additional work is required compared to an original construction contract. To be paid for the extra work, it is their responsibility to justify using supportive documentation ($A1$). Hence, the subcontractor submits a change management request on the platform ($A2$). Further, the TPM must notify the subcontractor that his request has been initiated ($A5$), as well as checking the request specifications against the initial contract requirements and the technical documentation ($A4$). Once the request is checked, the TPM can decide whether to accept the change request ($A7$), to reject the request ($A8$) or to ask for additional documents that support the subcontractors' claim ($A6$). If the TPM decides to reject the claim, she must attach reasoning for the decision and communicate it to the subcontractor. Next, the subcontractor can evaluate the rejection ($A16$). If there is need for further documentation to support the claim, the TPM must send a request for additional information ($A1$). If the TPM agrees with the change, she must forward documentation describing what changes from the initial contract to the

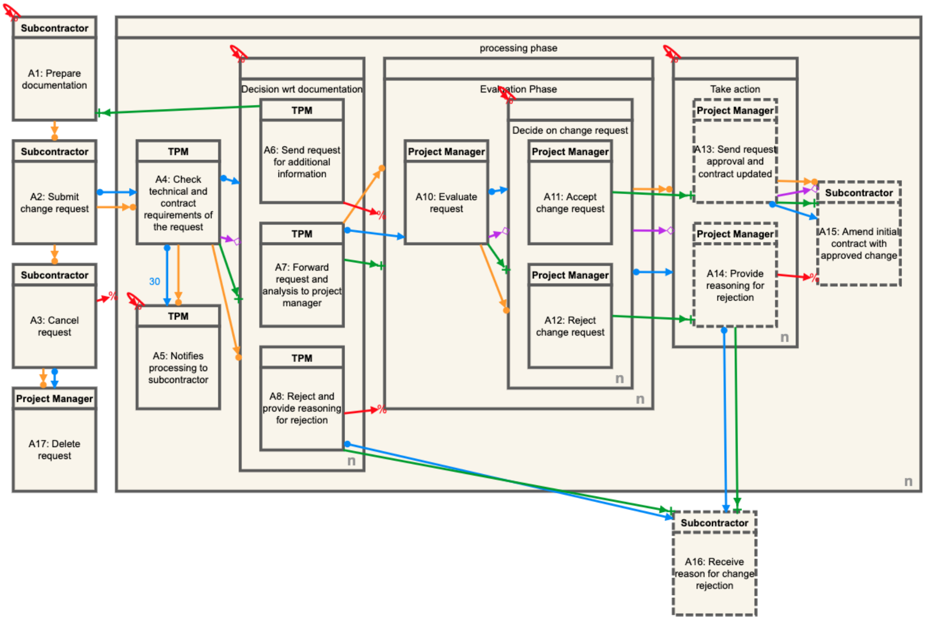

**Fig. 3.** Contract Change Management Process $P_{spec}$

project manager. The project manager must evaluate the request ($A10$). He is responsible for taking the final decision, whether to accept ($A11$) or reject ($A12$) the request. In case of rejection, the project manager must notify the subcontractor about the decision and substantiate with reasoning ($A14$). Besides, if the answer is an acceptance, the project manager is responsible for sending an updated contract form ($A13$). Once the new contract is received, the subcontractor must attach it to the old contract ($A15$). As part of the DMS capabilities, the subcontractor is allowed to cancel the change request ($A3$) at any point after submission, with the effects of deleting the application ($A17$).

The diagram in Fig. 3 provides a visual representation of process $P_{spec}$ described above[6]. Events are denoted via boxes, and arrows describe the relations introduced in the previous section. Each event has a label presenting its description, as well as the role of the agent(s) that can execute the event. An included event is represented with a solid border, with a dashed line if it is excluded. Included events can be executed at any time (unless they become excluded), and, unless preceded by a response relation, they can also be left unexecuted. Relations can point to events or to events "collections" (boxes marked with "n"). As formalised in [23], such collections are referred to as "nestings" and are just a visual shorthand, understanding arrows to (from) nestings to represent arrows to (from) every event inside the nesting.

[6] The process is available for simulation and execution at https://www.dcrgraphs.net/tool/main/Graph?id=43ea382d-de1b-4278-8eff-591426244d90

$$\frac{i \Rightarrow h \geq k}{[M, e : (h, i, \_), f : (\_, \mathsf{t}, \_)]\ e \xrightarrow{k}\!\bullet f \vdash f : (\emptyset, \emptyset, \emptyset)} \qquad \frac{}{[M, e : (\_, \mathsf{t}, \_)]\ e \bullet\!\xrightarrow{k} f \vdash e : (\emptyset, \emptyset, \{f : k\})}$$

$$\frac{i \Rightarrow (p = \mathsf{f})}{[M, e : (\_, i, p), f : (\_, \mathsf{t}, \_)]\ e \rightarrow\!\diamond f \vdash f : (\emptyset, \emptyset, \emptyset)} \qquad \frac{}{[M, e : (\_, \mathsf{t}, \_)]\ e \rightarrow\!+ f \vdash e : (\emptyset, \{f\}, \emptyset)}$$

$$\frac{}{[M, e : (\_, \mathsf{t}, \_)]\ e \rightarrow\!\% f \vdash e : (\{f\}, \emptyset, \emptyset)} \qquad \frac{}{[M, e : (\_, \mathsf{t}, \_)]\ 0 \vdash e : (\emptyset, \emptyset, \emptyset)}$$

$$\frac{e \neq f' \qquad \mathcal{R} \in \{\xrightarrow{k}\!\bullet, \rightarrow\!\diamond\}}{[M, e : (\_, \mathsf{t}, \_)]\ f\ \mathcal{R}\ f' \vdash e : (\emptyset, \emptyset, \emptyset)} \qquad \frac{e \neq f \qquad \mathcal{R} \in \{\bullet\!\xrightarrow{k}, \rightarrow\!+, \rightarrow\!\%\}}{[M, e : (\_, \mathsf{t}, \_)]\ f\ \mathcal{R}\ f' \vdash e : (\emptyset, \emptyset, \emptyset)}$$

$$\frac{[M]\ T_i \vdash e : (\mathsf{Ex}_i, \mathsf{In}_i, \mathsf{Pe}_i) \quad i = \{1, 2\}}{[M]\ T_1 \parallel T_2 \vdash e : (\mathsf{Ex}_1 \cup \mathsf{Ex}_2, \mathsf{In}_1 \cup \mathsf{In}_2, \mathsf{Pe}_1 \cup \mathsf{Pe}_2)}$$

**Fig. 4.** Enabling & effects. We write "_" for "don't care", i.e., either true $\mathsf{t}$ or false $\mathsf{f}$

We point to some of the behavioural aspects in the model. The condition relation between $A1$ and $A2$ forbids the subcontractor to perform a submission without documentation. The exclusion relation to itself in $A1$ says that such activity can be done once per case, and it will cease to be available until it is included again (via the execution of $A6$). The response between "Decide on change request" and "Take action" says that once the activities $A11$ or $A12$ have been performed, it is obligatory to execute the included activities in the take action part. Only one decision can be taken per round, as the execution of $A11$ and $A12$ exclude each other. The chain of milestones and responses between $A10$ and $A15$ ensures that the attached copy only corresponds to the most updated decision: every time a project manager executes $A10$, the activities inside "decide on change request" become pending. This will inhibit any action until the decision has been revised. Finally, the timed response between $A4$ and $A5$ says that notification must be done within 30 time units of the execution of $A4$.

### 3.1 Semantics

We first define when an event is *enabled* and what *effects* it has if executed. The judgement $[M]\ T \vdash e : (Exc, Inc, Pen)$, defined in Figure 4, should be read: "in the marking $M$, the term $T$ allows the event $e$ to happen, with the effects of excluding events $Exc$, including events $Inc$, and making events $Pen$ pending."

The first rule says that if $e$ is a condition for $f$, then $f$ can happen only if (1) it is itself included, and (2) if $e$ is included, then $e$ happened at least $k$ steps ago. The second rule says that if $e$ is a milestone for $f$, then $f$ can happen only if (1) it is itself included, and (2) if $e$ is included, then $e$ must not be pending. The third rule says that if $f$ is a response to $e$ and $e$ is included, then $e$ can happen with the effect of making $f$ pending with a deadline of $k$. The fourth (respectively fifth) rule says that if $f$ is included (respectively excluded) by $e$ and $e$ is included, then $e$ can happen with the effect of including (respectively excluding) $f$. The sixth rule says that for an unconstrained process 0, an event $e$ can happen if it is included. The seventh rule says that a relation allows any included event $e$ to happen without effects when $e$ is not the relation's right-hand–side event.

$$\frac{[M]\ T \vdash e : \delta}{T \vdash M \xrightarrow{e} \delta\langle e\langle M\rangle\rangle}\ [\textsc{Event}] \qquad \frac{\mathsf{deadline}\langle M\rangle > 0}{T \vdash M \xrightarrow{\mathsf{tick}} \mathsf{tick}\langle M\rangle}\ [\textsc{Time}]$$

**Fig. 5.** Transition semantics.

Finally, the last rule says that enabledness for parallel composition depends on its constituents (we omit symmetric rules for sake of clarity).

Given enabling and effects of events, we define the *action* of respectively an *event* $e$ and an *effect* $\delta = (\mathsf{Ex}, \mathsf{In}, \mathsf{Pe})$ on a marking $M$ pointwise by the action on individual event states $f : (h, i, r)$ as follows. Assume $e$ is enabled in the process $[M]\ T$ with effect $\delta = (\mathsf{Ex}, \mathsf{In}, \mathsf{Pe})$. The state of $e$ tracks that the event has happened now, setting its executed flag to 0. Similarly, we say that it is not longer pending. The effect of executing $e$ in a marking $M$, written $e\langle M\rangle$, is inductively defined as follows:

$$e\langle M\rangle = \begin{cases} \epsilon & \text{if } M = \epsilon \\ e\langle N\rangle, f : (0, i, \mathsf{f}) & \text{if } M = N, f : (\_, i, \_) \wedge e = f \\ e\langle N\rangle, f : (h, i, r) & \text{if } M = N, f : (h, i, r) \wedge e \neq f. \end{cases}$$

The application of effect $\delta = (\mathsf{Ex}, \mathsf{In}, \mathsf{Pe})$ over a marking $M$, denoted $\delta\langle M\rangle$, is inductively defined as follows:

$$\delta\langle M\rangle = \begin{cases} \epsilon & \text{if } M = \epsilon \\ \delta\langle N\rangle, f : \big(\, h,\ \underbrace{(i \wedge f \notin \mathsf{Ex}) \vee f \in \mathsf{In}}_{\text{included?}},\ r' \big) & \text{if } M = N, f : (h, i, r) \end{cases}$$

Where $r' = \mathsf{min}\{d \mid (f, d) \in \mathsf{Pe}\}$ if $(f, d) \in \mathsf{Pe}$ and $r' = r$ otherwise. That is, the event only stays included (second component) if $f \notin \mathsf{Ex}$ (it is not excluded) or $f \in \mathsf{In}$ (it is included). The pending flag takes the minimal deadline for which $f\colon d \in \mathsf{Pe}$, otherwise, it keeps the flag unchanged. Note that an event can be both excluded and included by the effect, conceptually the exclusion happens first, followed by the inclusion.

The transition semantics requires us to account for the time that has passed between events. The $\mathsf{deadline}$ function is inductively defined over markings:

$$\mathsf{deadline}\langle M\rangle = \begin{cases} \omega & \text{if } M = \epsilon \\ \mathsf{min}\{p', \mathsf{deadline}\langle M'\rangle\} & \text{if } M = M', e\colon (h, i, p) \end{cases}$$

With $p'$ taking the value of $p$ if $i = \mathsf{t}$, otherwise $p' = \omega$. Basically, only deadlines of included events are considered. The deadline function sets a lower limit for events to happen. Moreover, we need to update the marking by incrementing the time after an event has fired. The $\mathsf{tick}$ function is inductively defined over markings with such purpose:

$$\mathsf{tick}\langle M\rangle = \begin{cases} \epsilon & \text{if } M = \epsilon \\ \mathsf{tick}\langle M'\rangle, e\colon (h + 1, i, \mathsf{max}\{0, p - 1\}) & \text{if } M = M', e\colon (h, i, p) \end{cases}$$

Extending the $+$ and $-$ operators such that $\mathsf{f} + 1 = \mathsf{f}$ and $\mathsf{f} - 1 = \mathsf{f}$, and $\omega - 1 = \omega$.

Figure 5 introduces the transition semantics of processes. In rule [Event], the marking $M$ fires an enabled event $e$, generating as a result a marking $M'$.

Note that transitions are non-deterministic: more than one event can be enabled in $M$. In rule [TIME], the marking $M$ is updated in one unit, generating $M'$. Intuitively, a transition $T \vdash M \xrightarrow{e} M'$ expresses that process $[M]\ \lambda T$ fires an event $e$ and modifies its marking to $M'$. As customary, we denote with $\xrightarrow{e}^*$ the transitive closure of $\xrightarrow{e}$. Moreover, we define the state space of $[M]\ T$ as $\mathcal{P}([M]\ T) = \{[M']\ T \mid T \vdash M \xrightarrow{e}^* M'\}$. Event transitions give rise to a labelled transition system $lts([M]\ \lambda T) = \langle \mathcal{P}(M), [M]\ T, \mathcal{E}', \longrightarrow, \Sigma, \lambda' \rangle$, where $[M]\ T \in \mathcal{P}([M]\ T)$ is the initial state, $\mathcal{E}' = \mathcal{E} \cup \{\mathsf{tick}\}$ is the set of labels, $\longrightarrow \subseteq \mathcal{P}([M]\ T) \times \mathcal{E}' \times \mathcal{P}([M]\ T)$, $\Sigma$ is an alphabet, and a labelling function $\lambda' \subseteq \mathcal{E} \times \Sigma$ defined by $\lambda(e) = \lambda(e)$ for $e \in \mathcal{E}$, and $\lambda'(\mathsf{tick}) = \mathsf{tick}$.

We equip with the LTS with notions of *accepting runs*, incorporating similar notions defined for DCR Graphs [6,32] to their timed setting:

**Definition 1 (Runs, Accepting Runs).** *A run of* $[M]\ T$ *is a finite or infinite sequence of transitions* $[M]\ T = [M_0]\ T_0 \to e_0 \cdots$ . *A run is* accepting *iff for every state* $[M_i]\ T_i$*, when* $M_i(e) = (\_, \mathsf{t}, \mathsf{t})$ *then there exists* $j \geq i$ *s.t. either* $M_j(e) = (\_, \mathsf{f}, \_)$ *or* $[M_j]\ T_j \xrightarrow{e} [M_{j+1}]\ T_{j+1}$.

In other words, an accepting run consider transitions that either execute pending events, or excludes them. Note that since an event $e$ may happen more than once, even processes with only finitely many events may have infinite runs. Having defined the LTS and runs we can define the language defined by a DCR process to be its set of accepting runs.

**Definition 2 (Traces).** *A* trace *of a process* $[M]\ \lambda T$ *is a possibly infinite string* $s = (s_i)_{i \in I}$ *s.t.* $[M]\ T$ *has an accepting run* $[M_i]\ T_i \xrightarrow{e_i} [M_{i+1}]\ T_{i+1}$ *with* $s_i = \lambda(e_i)$. *Finally, the process* $[M]\ T$ *has the* language $\mathsf{lang}([M]\ \lambda T) = \{s \mid s \text{ is a trace of } [M]\ \lambda T\}$.

## 4 Compliance Rules

Not all law paragraphs are created equal. Different articles describe definitions, commencement periods, amendments, and other provisions. We focus on *self-contained* procedural articles, those paragraphs that do not depend on the state of affairs of events described in other paragraphs. One example is GDPR Art. 21 §1:

> *(Right to Object) §1. The data subject shall have the right to object, on grounds relating to his or her particular situation, at any time to processing of personal data concerning him or her [...]. The controller shall no longer process the personal data unless the controller demonstrates compelling legitimate grounds for the processing which override the interests, rights and freedoms of the data subject or for the establishment, exercise or defence of legal claims.*

| Legal Text | Policy | Compliance Rule |
|---|---|---|
| GDPR Art. 21 §1. | If the subcontractor submits a change request, he may cancel it afterwards. After cancellation, the project manager must eventually delete the request. | $RC1 = [e_1 : (\mathsf{f},\mathsf{t},\mathsf{f}), e_2 : (\mathsf{f},\mathsf{t},\mathsf{f}), e_3 : (\mathsf{f},\mathsf{t},\mathsf{f})]\ \lambda_1 T_1$<br>$T_1 = e_1 \rightarrow\!\bullet e_2 \parallel e_2 \rightarrow\!\bullet e_3 \parallel e_2 \bullet\!\rightarrow e_3$<br>$\lambda_1(e_1)$ = "A2: submit a change request"<br>$\lambda_1(e_2)$ = "A3: cancel change request"<br>$\lambda_1(e_3)$ = "A17: delete the request" |
| 95/46/EC. Sect IV, Art. 11. §1. [. . . ] The controller [. . . ] must at the time of undertaking the recording of personal data [. . . ] provide the data subject with at least the following information [. . . ]. | After the subcontractor submits a change request, eventually the TPM will notify the subcontractor about the processing of request, including the personal data used. | $RC2 = [e_4 : (\mathsf{f},\mathsf{t},\mathsf{f}), e_5 : (\mathsf{f},\mathsf{t},\mathsf{f})]\ \lambda_2 T_2$<br>$T_2 = e_4 \bullet\!\rightarrow e_5 \parallel e_4 \rightarrow\!\bullet e_5$<br>$\lambda_2(e_4)$ = "A2: Submit change request<br>$\lambda_2(e_5)$ = "A5: Notifies processing to subcontractor" |
| Organization KPI. A change request should take a maximum amount of time, otherwise it becomes invalid. | The change request is valid for 60 working days and afterwards it is closed. | $RC3 = [e_6 : (\mathsf{f},\mathsf{t},\mathsf{f}), e_7 : (\mathsf{f},\mathsf{f},\mathsf{f}), e_8 : (\mathsf{f},\mathsf{t},\mathsf{f})]\ \lambda_3 T_3$<br>$T_3 = e_6 \rightarrow\!+ e_7 \parallel e_6 \bullet\!\xrightarrow{60} e_7 \parallel e_6 \xrightarrow{60}\!\bullet e_8 \parallel e_8 \rightarrow\!\% e_7$<br>$\lambda_3(e_6)$ = "A2: Submit change request"<br>$\lambda_3(e_7)$ = "Finish Processing request"<br>$\lambda_3(e_8)$ = "Cancel Processing" |

**Fig. 7.** Elicitation of Compliance Rules

We observe dependencies between two events, ($B_1$) processing of personal data, and ($B_2$) the right to object. We also observe the consequences of applying $B_2$. For the sake of clarity we assume "no longer process personal data" as the event ($B_3$) "stop processing". The process for Art. 21 §1 is:

| Event in Legislation | Activity/event in Process Model |
|---|---|
| $B_1$: Process personal data | A2: Submit change request |
| $B_2$: Right to object | A3: Cancel request |
| $B_3$: Stop processing | A17: Delete request |

**Fig. 6.** Instantiation of Art 21. GDPR for process in Fig. 3

$$RF_1 = [B_1 : (\mathsf{f},\mathsf{t},\mathsf{f}), B_2 : (\mathsf{f},\mathsf{t},\mathsf{f}), B_3 : (\mathsf{f},\mathsf{t},\mathsf{f})]\ B_1 \rightarrow\!\bullet B_2 \parallel B_2 \bullet\!\rightarrow B_3$$

The reference model requires a mapping from abstract rights such as "right to object" into activities/events in the business process. Further knowledge from implementation guidelines is used to determine the proper mapping for concepts such as "data subject", "controller" or "personal data". Fig. 6 presents a mapping between events Art. 21 §1 and and events in $P_{spec}$ in Fig. 3.

The result of combining the dependencies from laws and business process information gives rise to compliance policies that are specific to the domain. A natural language policy such as *"in case (the subcontractor) submits a change request, (the subcontractor) may cancel the change request. If (the subcontractor) cancels the request, (the project manager) must eventually delete the request"*. These policies are formalized in terms of DCR processes. Fig. 7 present some exemplary policies. We will refer as *compliance rules* to the resulting DCR processes in this stage.

We capture event dependencies by relying on test-driven development [42, 46], which serves as means of validation when introducing constraints in the model. Interestingly, test-driven development aligns with current practices when introducing changes in a law. Scenarios correspond to legal precedents [27]. In common law, a legal precedent corresponds to a previous case that establishes a principle or rule. This principle is then used by judicial bodies when deciding later cases with similar issues or facts. Compliance rules can be tested against scenarios representing legal precedents, where valid rules should at least be able to reach the same decisions from earlier precedents.

The last step in the elicitation of compliance rules is the alignment between the compliance rules and the process model.

**Definition 3 (Term Alignment & Target events).** *Let $L, L' \subseteq \mathcal{L}$.*
*A* term alignment *is the total function $g : L \to L'$. If $P, Q$ are DCR processes with labels $L, L'$ respectively, we say that $g$ is a term alignment from $P$ to $Q$ if $g$ is a term alignment from $L$ to $L'$. Moreover, we define the* target events *of $g$ for $e$ in $P$ as $tg(g, e, P) = \lambda^{-1}(g(\lambda(e)))$.*

Although term alignment is an arbitrary function defined by the compliance officer, we require for simplicity of the exposition that there is exactly a *single* target event for each event.

Note that more than one $g$ can be defined if the rules in the law applies to more than one set of events in the process. Also, $g$ will typically be non-surjective since the business process might contain activities that do not map to any legal requirement.

**Definition 4 (Instances of a Compliance Rule).** *Let $G = \{g_1, \ldots, g_n\}$ be a set of term alignments from $P$ to $Q$. An* instance of $P$ under $g$ in $Q$, *written $P{\downarrow_g}Q$ for $g \in G$, is $P\sigma$ with labelling $\lambda'(e) = g(\lambda(e))$, such that $\sigma = \{f_1, \ldots, f_n / e_1, \ldots, e_n\}$ where $f_i = tg(g, e_i, P)$. We denote by $Inst(P, G, Q) = \{P{\downarrow_g}Q \mid g \in G\}$ the set of all instances of $P$ under $G$ in $Q$.*

*Example 4.2.* The term alignments $g_1, g_2$ are built from the obvious maps from events in $RC1$ and $RC2$ to events with same labels in $P_{spec}$ in Fig. 3. Two term alignments are required for $RC3$:

| **Term Alignment** | **Label Reference Model** | **Event** $P_{spec}$ | **Label Process Model** |
|---|---|---|---|
| $g_3$ | A2: submit a change request | $f_1$ | A2: submit a change request |
| | Finish Processing request | $f_2$ | A15: Amend initial contract |
| | Cancel Processing | $f_3$ | A3: Delete request |
| $g_4$ | A2: submit a change request | $f_1$ | A2: submit a change request |
| | Finish Processing request | $f_4$ | A16: Receive reason for change rejection |
| | Cancel Processing | $f_3$ | A15: Delete request |

The set of term alignments for each compliance rule is respectively $G_1 = \{g_1\}, G_2 = \{g_2\}$, and $G_3 = \{g_3, g_4\}$. As can be seen from Def. 4, the set of instances substitute the events for the corresponding ones in $P_{spec}$, so

$$Inst(RC3, G_3, P_{spec}) =$$

$$\left\{ \begin{array}{l} [f_1 : (\mathsf{f},\mathsf{t},\mathsf{f}), f_2 : (\mathsf{f},\mathsf{f},\mathsf{f}), f_3 : (\mathsf{f},\mathsf{t},\mathsf{f})]\ \lambda_3 f_1 {\to}{+} f_2 \parallel f_1 {\bullet}\!\xrightarrow{60} f_2 \parallel f_1 \xrightarrow{60}\!{\bullet} f_3 \parallel f_3 {\to}\% f_2, \\ [f_1 : (\mathsf{f},\mathsf{t},\mathsf{f}), f_4 : (\mathsf{f},\mathsf{f},\mathsf{f}), f_3 : (\mathsf{f},\mathsf{t},\mathsf{f})]\ \lambda_3 f_1 {\to}{+} f_4 \parallel f_1 {\bullet}\!\xrightarrow{60} f_4 \parallel f_1 \xrightarrow{60}\!{\bullet} f_3 \parallel f_3 {\to}\% f_4 \end{array} \right\}$$

Moreover, labels have also changed, being $\lambda_3(f_2) =$ "A15: Amend initial contract with approved change", and $\lambda_3(f_4) =$ "A16: Receive reason for change rejection".

## 5 Compliance Checking by Refinement

In previous sections we showed how to use DCR processes for the specification of declarative workflows (c.f. Section 3), and the generation of compliance rules (c.f.: Section 4). In this section, we will consider compliance as a particular instance of DCR process refinement [10], between each of the instances generated by a compliance rule, and the process specification.

Abstractly, we take refinement to be just inclusion of languages (trace sets). Given a sequence $s$, write $s_i$ for the $i$-th element of $s$, and $s|_\Sigma$ for the largest sub-sequence $s'$ of $s$ such that $s'_i \in \Sigma$ for $0 < i \leq |s|$; e.g, if $s = AABC$ then $s|_{A,C} = AAC$. We lift projection to sets of sequences point-wise.

**Definition 5 (Refinement [11]).** *Let $P, Q$ be processes. We say that $Q$ is a* refinement *of $P$ iff* $\mathsf{lang}(Q)|_{\mathsf{alph}(P)} \subseteq \mathsf{lang}(P)$. *We will write $R \sqsubseteq P$ whenever $R$ is a refinement of $P$.*

In practice, we will use a notion of refinement by composition, as introduced in [11] to define a "refines" relation between a process and an instance of a compliance rule. To define composition, we need to merge parallel markings and effects. Merge on markings is *partial*, since it is only defined on markings that agree on their overlap:

$$\begin{aligned}(M_1, e:m) \oplus (M_2, e:m) &= (M_1 \oplus M_2), e:m \\ (M_1, e:m) \oplus M_2 &= (M_1 \oplus M_2), e:m \quad \text{when } e \notin \mathsf{dom}(M_2) \\ M_1 \oplus (M_2, e:m) &= (M_1 \oplus M_2), e:m \quad \text{when } e \notin \mathsf{dom}(M_1).\end{aligned}$$

The *merge of effects* $\delta$ is defined as the pointwise union of each of the sets of excluded/included/pending events: $(Exc_1, Inc_1, Pen_1) \oplus (Exc_2, Inc_2, Pen_2) = (Exc_1 \cup Exc_2, Inc_1 \cup Inc_2, Pen_1 \cup Pen_2)$.

**Definition 6 (Merge & Marking Compatibility).** *The merge of processes $[M]\ \lambda_1 T$ and $[N]\ \lambda_2 U$ is defined if the merge of markings $M \oplus N$ is defined and the labelling functions agree as well, in which case $[M]\ \lambda_1 T \oplus [N]\ \lambda_2 U = [M \oplus N]\ (\lambda_1 \cup \lambda_2)\,(T \parallel U)$. If the merge of two processes is defined, we say that they are* marking compatible.

We can now define the refines relation between an instance $P$ of a compliance rule and a marking compatible process $Q$ (i.e.: the process model) as follows.

**Definition 7 (Refines).** *Let $P, Q$ be marking compatible processes. We say that $Q$* refines *$P$ iff $P \oplus Q \sqsubseteq P$.*

Note that even though $P \oplus Q = Q \oplus P$, it may still be the case that $P \oplus Q \sqsubseteq P$ but not of $P \oplus Q \not\sqsubseteq Q$.

**Definition 8 (Compliance).** *Let $P, R$ be DCR processes, and $G$ be a set of term alignments from $R$ to $P$. We say that $P$* is strongly (resp. weakly) compliant *with $R$ under $G$, written $P \leq^s_G R$ (resp. $P \leq^w_G R$) if $\forall R_i \in Inst(R, G, P)$, $P$ refines $R_i$ (resp. if $\exists R_i \in Inst(R, G, P)$, $P$ refines $R_i$).*

That is, take rule $R$, a process $P$ and a term alignment mapping labels in $R$ to $P$. (Strong) compliance requires us to 1) generate all instances of $R$ in $P$ and 2) check whether the merge of each instance with the $P$ is compatible (i.e. refines) the instance. Notice that while instances and the process will have their merge defined, $P$ might have different constraints that might affect refinement.

We close this section stating results regarding the decidability and tractability of compliance checking for DCR processes.

**Theorem 1 (Compliance checking is decidable).** *Let $P, R$ be DCR processes, and let $G$ be a set of term alignments from $R$ to $P$. Then checking $P \leq^{w}_{G} R$ and $P \leq^{s}_{G} R$ is decidable.*

*Proof.* We know from [11] that refinement of DCR processes is known to be decidable; this fact relies on the state space of a DCR process being finite. Time does not change this; see [24] for details. It is therefore sufficient to prove that for any $R$ and $G$, the set $Inst(R, G, P)$ is finite. By Definition 3, this set is bounded by the size of $G$ and the number of possible substitutions $\sigma$. But $G$ is finite by definition, and $\sigma$ is clearly uniquely determined given a $g \in G$. □

While generally checking refinement for DCR processes is NP-hard already in the absence of time, [11] showed that the refines relation can be approximated by a static property, the non-invasiveness on the graphs recalled below.

**Definition 9 (Non-invasiveness [11]).** *Let $P = [M_P]\ \lambda_P\, T_P$ and $R$ be marking compatible processes. We say that $P$ is* non-invasive *for $R$ iff*

1. *For every context $C[-]$, such that $T_P = C[e \rightarrow\!\% \ f]$ or $T_P = C[e \rightarrow\!+\ f]$, $f \notin \mathsf{fe}(R)$; and*
2. *For every label $l \in \mathsf{alph}(P) \cap \mathsf{alph}(R)$, if $e \in \mathsf{fe}(P)$ is labelled $l$, then $e \in \mathsf{fe}(R)$.*

.

That is, a process $P$ is non-invasive for a process $R$ if it does not introduce inclusion or exclusion relations on the events of $R$. We note that this property can straightforwardly be determined in polynomial time.

**Lemma 1.** *Non-invasiveness is decidable in polynomial time.*

*Proof.* Follows from Definition 9: an algorithm only needs to check for each inclusion and exclusion relation in $P$ if the target event exists in $R$.

In [11] it was also shown that non-invasiveness guarantees the refine relation. This can be extended to timed processes.

**Theorem 2.** *If $P$ is non-invasive for $R$ then $P$ refines $R$.*

*Proof (sketch).* We need to extend the proof in [11] to timed processes observing the following: 1) in the case of conflicting deadlines the most strict deadlines always take precedence, 2) therefore after composition of a $R$ and $P$ which share a timed relation with a different deadline, the most strict deadline will be followed, and 3) the composed process will not allow for traces which were forbidden under the strictest deadline. □

We can apply this result to compliance, and show that a process is compliant with a compliance rule, if it is non-invasive for all term alignments.

**Lemma 2.** *Let $P, R$ be DCR processes, and $G$ be a set of term alignments from $R$ to $P$, $P$* is strongly (resp. weakly) compliant *with $R$ under $G$ if $\forall R_i \in Inst(R, G, P)$, $P$ is non-invasive for $R_i$.*

*Proof.* Follows directly from Definition 8 and Theorem 2.

Correspondingly, this means that compliance checking is a polynomial time task if $P$ is non-invasive for $R$ for all term alignments.

**Theorem 3.** *If $P$ is non-invasive $\forall R_i \in Inst(R, G, P)$, then checking $P \leq^{w}_{G} R$ and $P \leq^{s}_{G} R$ is polynomial in $R, G, P$.*

*Proof.* Follows directly from Lemmas 1 and 2.

We conclude that through careful construction of the process model, in particular by avoiding the unnecessary introduction of exclusion and inclusion relations on events which may be governed by compliance rules, we can significantly reduce the time complexity of checking the compliance of the process. This comes in contrast to approaches based in annotated imperative business processes, which to a great extent belong to the non-polynomial complexity class [45].

**Corollary 1.** *$P_{spec} \leq_{G_1} RC1$, $P_{spec} \leq_{G_2} RC2$, and $P_{spec} \not\leq_{G_3} RC3$*

## 6 Adoption considerations

We describe two uses of the compliance framework: one at the municipality of Syddjurs (DK), and another at the municipality of Genoa (IT). The municipalities selected processes in different domains: the provision of benefits offered to young persons with special needs (DK), and the release of construction permits (IT). They were regulated by different laws, for which reference models of selected articles were created by compliance specialists. The reference models of articles in the Danish Consolidation for Social Services [44] and the Construction Law of the Liguria region [40] vary on size and complexity, ranging from a minimum of 4 events and 12 relations, up to 86 events and 125 relations in a single article. The intended use of the framework varied: while Syddjurs aims at driving a new implementation of their processes, Genoa wanted to verify their current implementations with respect to the law. The work was carried out by case workers within the municipality (DK), and a consultancy house (IT). We collected feedback from users generating reference models of law about their use, benefits and challenges. Both organisations commented that the pairing of laws and models provide them traceability, and allowed lawyers to be part in the co-creation of process implementations using their domain knowledge. Moreover, law-process pairings helped them to understand the legislation, making evident bottlenecks in a process (an activity that for which many other events depend on), and showed them previously unknown paths for achieving goal, while still be in accordance to the law. This aligns with previous studies on comprehension of hybrid artefacts combining texts and declarative models [3]. On their use, both organisations agreed that some laws are too general, and they required implementation guidelines to complete their models. A challenge concerned the writing style of the guidelines: if guidelines have been written in an imperative style, there is a risk of over-constraining the model. When asked about the understandability of the models, they reported that after an initial training, generated models were understandable for compliance specialist, and they could be used as communication artefacts. However, they also reported challenges on the understandability of large models, and suggested the inclusion of abstractions to increase model comprehension. With respect to compliance, the main challenge concerned term alignment, as it currently needs to be hard-coded (no tool support). In some cases, an event in the law had a 1-to-many correspondence with the process. Another suggestion was to extend feedback support to reasons for non-compliance, rather than yes/no outputs.

## 7 Related Work

We can divide related approaches into four categories:

*Model Checking techniques*: Most model checking techniques for compliance [19] represent the process as a finite state machine and the laws in a temporal logic. We differ from such approaches in that we use a declarative process language both for defining the process and laws. The reasons are threefold: First, it is known that some of these languages present technical difficulties when modelling permissions, obligations and defeasible (i.e.: exceptional) conditions [16]. These concepts are straightforward in DCR graphs: permissions are encoded as enabled events, obligations are the composition of events using a response relation, and defeasible conditions are represented by mutual exclusion relations between events. The second advantage is the possibility of combining process narratives and visual notations: our work puts forward the recommendations from [36] that states that higher cognitive loads can be achieved when combining process descriptions with graphical notations. This is particularly important in our case, as compliance specialists in local governments do not have prior training in using verification techniques using temporal logics. Finally, verification is efficient: it relies on refinement of transition systems with responses [6,28], and although the complexity process refinement belongs to the category of NP-hard problems [11], we have shown that we can use syntactic restrictions to check compliance in polynomial time.

*Compliance Refinement*: Seaflows [31] proposes an alignment of compliance requirements into business processes. Laws are modelled in terms of constraints over event traces that can be verified at design-time and monitored at run-time. However, no specific constraint specification language is provided. The work in [41] presents a refinement-based approach where abstract business processes representing laws are incrementally refined until executable processes can be generated. The nature of such abstract business processes is imperative, given in BPMN diagrams, which imposes rigidity on how to achieve certain rights.

*Compliance-by-design (CbD)*: FCL/PCL & Regorous [13,14,17,18,21] treats compliance as a property of the process to execute while not violating the laws in a regulation. Compliance checking requires to 1. identify the deontic effects of the set of modelled regulations, 2. determine the tasks and the obligations in force for each task, and 3. check whether the obligations have been fulfilled or postponed after the execution of a task. While we subscribe to CbD as a methodology, our approach differs in the fact that there is no need to map a declarative language (such as PCL and FCL) into an imperative specification.

*Visual Languages for Compliance:* The work in [26] introduces eCRG, a visual modelling notation for compliance rules including control flow, interaction, time, data, and resource perspectives. eCRG rules are then paired with event logs to determine whether completed or running process instances are compliant. While our approach is mostly tailored to design stages, [26] focuses on after-the-fact compliance. Finally, the BPMN-Q language [4] provides a visual notation to CTL, and the language describes compliance rules including control and data flow aspects, that are later model-checked against BPMN models. Declare [38] is LTL based and in principle, the compliance checking approach presented here could also be used. However, its LTL-semantics has been shown to present technical difficulties when modelling obligations and defeasible conditions [16].

## 8 Concluding Remarks

We presented a verification framework for the design of process models that are compliant with regulations. This work exploits the similarities of declarative process languages with logical languages to be able to express models of law. In this manner, both process models and models of law are described in the same declarative notation, and it becomes straightforward to verify whether compliance is achievable. We show that compliance can be checked efficiently in polynomial time, given careful construction of the models.

While the focus of this paper is centred on CbD approaches, it accommodates after-the-fact compliance. In future work we will explore other variants of compliance, such as process conformance based on event logs. Our results rely on the choice of DCR as language for reference and process models, and in this paper we have restricted ourselves to a version of DCR graphs without subprocesses and locality. The decidability results in Thm. 1 will not hold with the inclusion of these operators. We have not needed to consider such constructs in the construction of compliance rules so far, but it would be interesting to revisit them in future work, as well as multi-dimensional compliance policies [39].

**Acknowledgments:** Thanks to Nicklas Healy from Syddjurs Kommune, and Paolo Gangemi from MAPS Group for their evaluations on the compliance framework. This work has been financially supported by the Innovation Fund Denmark project EcoKnow.org (7050-00034A), and the European Union Marie Sklodowska-Curie grant agreement BehAPI No.778233.
